# *In vivo* expression of peptidylarginine deiminase in *Drosophila melanogaster*

**DOI:** 10.1371/journal.pone.0227822

**Published:** 2020-01-15

**Authors:** Olena Mahneva, Monica G. Risley, Ciny John, Sarah L. Milton, Ken Dawson-Scully, William W. Ja

**Affiliations:** 1 Department of Biological Sciences, Florida Atlantic University, Boca Raton, Florida, United States of America; 2 International Max Planck Research School (IMPRS) for Brain and Behavior, Boca Raton, Florida, United States of America; 3 Department of Neuroscience, The Scripps Research Institute, Jupiter, Florida, United States of America; 4 Center on Aging, The Scripps Research Institute, Jupiter, Florida, United States of America; Biomedical Sciences Research Center Alexander Fleming, GREECE

## Abstract

Peptidylarginine deiminase (PAD) modifies peptidylarginine and converts it to peptidylcitrulline in the presence of elevated calcium. Protein modification can lead to severe changes in protein structure and function, and aberrant PAD activity is linked to human pathologies. While PAD homologs have been discovered in vertebrates—as well as in protozoa, fungi, and bacteria—none have been identified in *Drosophila melanogaster*, a simple and widely used animal model for human diseases. Here, we describe the development of a human PAD overexpression model in *Drosophila*. We established fly lines harboring human PAD2 or PAD4 transgenes for ectopic expression under control of the GAL4/UAS system. We show that ubiquitous or nervous system expression of PAD2 or PAD4 have minimal impact on fly lifespan, fecundity, and the response to acute heat stress. Although we did not detect citrullinated proteins in fly homogenates, fly-expressed PAD4—but not PAD2—was active *in vitro* upon Ca^2+^ supplementation. The transgenic fly lines may be valuable in future efforts to develop animal models of PAD-related disorders and for investigating the biochemistry and regulation of PAD function.

## Introduction

Peptidylarginine deiminase (PAD, EC 3.5.3.15) catalyzes the deimination, or citrullination, of peptidylarginine to peptidylcitrulline in the presence of elevated Ca^2+^ [1,2]. Protein citrullination plays an important role in a number of physiological processes. For example, PAD4 acts as a corepressor to regulate expression of histone-associated genes [3,4]. In mammals, there are five highly conserved PADs, PAD1-4 and PAD6 [5]. While PAD homologs have been identified in vertebrates including fish and avian species [6–11]—as well as in protozoa, fungi, and bacteria [12–17]—there are no known PAD homologs in *Drosophila melanogaster*. PADs play important roles in physiological processes such as gene regulation [18,19]; formation of exosomes and microvesicles [20], which allow parasites to evade host immunity [21]; keratinization of skin [22,23]; and regulation of brain plasticity [24,25].

Conversely, aberrant or misregulated PAD activity is associated with disease—the loss of charge due to PAD modification can induce protein misfolding and other functional changes. PAD hyperactivity has been linked to severe human disease phenotypes including cancer [26], rheumatoid arthritis [27], multiple sclerosis [28,29], Alzheimer’s [30,31], and prion disease [32,33]. Models where PADs are genetically and not pathologically overexpressed in specific tissues of an organism may help elucidate the roles of these enzymes in disease etiology. In a transgenic mammalian model, the introduction of 30 copies of PAD2 cDNA in myelin sheath led to spontaneous signs of demyelination in the central nervous system, which is a hallmark of multiple sclerosis [34]. Interestingly, PAD2 overexpression in mouse retina resulted in increased facilitated neuronal transport and visual function restoration [35]. Previous work also showed that PAD2 overexpression in transgenic mice models led to spontaneous skin neoplasia formation as well as increased malignancy and tumor-related inflammation of chemically-induced tumors [36,37]. Although mouse models of PAD-related diseases have been developed, simple genetically tractable models for rapid screening or testing hypotheses have yet to emerge.

*D*. *melanogaster* has been previously used as an ectopic expression system for human disease genes, including Alzheimer’s amyloid-beta peptide [38,39] and prion protein encoded by the *PRNP* gene [40,41]. To develop a fly model of PAD-related diseases, we generated transgenic flies for *in vivo* expression of human PAD2 and PAD4. Surprisingly, we found that fly lifespan and reproduction were not significantly altered with PAD expression. The behavioral response to acute heat stress, which we speculated might enhance in vivo PAD activity, was also unaffected. These negative results were associated with a lack of detectable citrullination activity *in vivo*, although PAD4 activity could be induced *in vitro*. Our findings suggest that PAD expression in the fly is not directly harmful and the transgenic PAD fly model may provide a useful system for probing mechanisms of enzyme activation in future studies.

## Materials and methods

### Flies

Flies were developed on a standard medium composed of 1.2% sucrose, 3.1% active yeast, 5.8% cornmeal, and 0.75% agar (all w/v), supplemented with 1% orthophosphoric acid (v/v) and 1% methylparaben mix (22.2% methylparaben, w/v, in ethanol). Flies were maintained at 25°C under a 12-/12-hour light/dark cycle and ~70% relative humidity until used for experiments. P-element transformants containing human PAD2 or PAD4 cDNA sequences were generated in *w*^*1118*^ flies using standard techniques [42]. Briefly, cDNA was directionally cloned into the pUAST vector to place the ORF under the control of upstream activating sequences for the GAL4/UAS expression system [43]. After DNA sequencing to validate the cloned ORFs, the vectors were injected into *w*^*1118*^ embryos using a commercial service (Rainbow Transgenic Flies, Camarillo, CA). Independent transformants were selected by eye color and established as stable lines using appropriate genetic balancers. Transgenic lines (and other stocks including *da*-GAL4 and *elav*-GAL4) were outcrossed for at least 10 generations with *w*^*1118*^ prior to being used in experiments. *w*^*1118*^ is a common, inbred laboratory strain that has been maintained independently in our laboratory for over 9 years. Unless specifically stated, PAD2_2 and PAD4_7 were the lines used for PAD2 and PAD4 expression, respectively. Experimental lines were compared to controls harboring only the GAL4 driver or PAD transgene, which were generated by crossing the appropriate line with *w*^*1118*^.

### Lifespan

Survival studies were performed on single-sex cohorts as described previously [44]. Briefly, 0–2 day old flies were collected and maintained on fresh stock food (standard medium). After 2 days, these mixed-sex groups were anesthetized by CO_2_ and randomly allocated to food vials as single-sex cohorts. For the lifespan study, flies were transferred to fresh food vials and dead flies were scored every 2 days until the end of the experiment, when no flies remained alive. For each fly line, 1–3 vials of ~20 flies/vial were typically assessed in a single trial, and all deaths were pooled to generate each survival curve.

### Fecundity

Virgin males and females were collected on CO_2_ within 4–6 hours of eclosion and maintained in same-sex groups until the start of the experiment. At ~5 days of age, groups of 4 males + 4 females were CO_2_-anesthetized and co-housed. After recovering and mating over 24 hours, flies were transferred to fresh vials to begin the experimental egg laying period. Flies were transferred to fresh vials every 24 hours to obtain 3 days of egg lay counts. Vials with embryos were subsequently maintained at 25°C for ~2 weeks to assess total adult progeny and male/female ratios. Reciprocal crosses were tested using experimental flies and Canton-S as the control. For each cross, 2–3 vials of 4 males + 4 females per vial were typically assessed.

### Heat stress

Fly locomotor activity was assessed during and after exposure to heat stress using the *Drosophila* Activity Monitor (DAM, TriKinetics, Inc., Waltham, MA). Male flies (5–9 days old) were loaded individually into standard DAM tubes (5 mm × 65 mm). In the DAM system, infrared light bisects each tube perpendicular to its axis and fly activity is quantified by the number of beam breaks that are made. After acclimating flies in a DAM at room temperature (23°C) for 10 minutes, tubes were rapidly transferred to a 2^nd^ preheated DAM in a 40.5°C incubator. After 12.5 minutes, tubes were moved back to the room temperature DAM for an additional 2 hours of activity recording. During heat treatment, the last recorded time that the fly crossed the beam was scored as the time to locomotor failure. Recovery was scored as the time required for the first beam break after heat treatment. Flies that did not fail in the 12.5 minutes of heat treatment (<7% of the animals) were excluded from analysis. For each genotype, >20 individually housed flies were typically assessed.

### Bacterial transformation and protein extraction

Competent *E*. *coli* [Rosetta (DE3) or BL21] were transformed using a standard CaCl_2_ protocol with bacterial expression vectors (pET-16b or pGEX-6P-1) harboring human PAD2 or PAD4 cDNA, respectively. These vectors encode N-terminal tagged fusion proteins—hexahistidine followed by a Factor Xa site and GST followed by a PreScission protease site for PAD2 and PAD4, respectively. Transformed and non-transformed (control) bacteria were inoculated into 50 mL of Luria broth and incubated at 37 ºC overnight with shaking at 150 RPM. Bacteria were collected by centrifugation and lysed with lysozyme (0.66 mg/mL final) at 37 ºC for 30 minutes followed by manual homogenization in PBS-T extraction buffer (1× PBS, 0.1% Tween-20, 2 mM EDTA, 2 mM DTT, 1 × SigmaFAST^™^ protease inhibitor cocktail (Sigma-Aldrich, St. Louis, MO), pH 7.4). The resulting bacterial lysates were spun at 13,200 RPM (Eppendorf 5415R, F45-24-11 rotor, Eppendorf, Hamburg, Germany) at 4 ºC for 10 minutes and the supernatants were saved. Protein concentration was determined using the Pierce 660 nm Protein Assay kit (ThermoFisher, Waltham, MA), following the manufacturer’s instructions. GST from the PAD4 recombinant fusion protein was cleaved using PreScission Protease (GE Healthcare, Chicago IL) according to the manufacturer’s directions.

### Protein extraction from *Drosophila* larvae and adults

To extract protein from *Drosophila* larvae and adults, ~20 3^rd^ instar larvae or adult males (7–9 days old) were collected, flash frozen, and homogenized in PBS-T extraction buffer in a 1.7-mL centrifuge tube using a plastic pestle homogenizer. The homogenates were centrifuged at 13,200 RPM (Eppendorf 5415R, F45-24-11 rotor) at 4 ºC for 10 minutes and the supernatants were collected and stored at -80 ºC. Protein concentration was determined as above.

### SDS-PAGE and Western blots

For protein electrophoresis, equivalent total protein was loaded into each lane of a 4–12% SDS-PAGE gel and run under reducing conditions. Resolved proteins were transferred to nitrocellulose membrane (GE Healthcare, Little Chalfont, United Kingdom), which was then blocked in 5% milk/TBST followed by overnight incubation in 5% milk/TBST supplemented with anti-PAD4 (ThermoFisher, Waltham, MA) or anti-PAD2 (Abgent, San Diego, California) antibody at 1:3000 or 1:1000 dilution, respectively. Membranes were washed in TBST and incubated for 1 hour in 5% milk/TBST buffer containing 1:4000 dilution of goat anti-rabbit HRP-conjugated secondary antibody (Abcam, Cambridge, UK). After final washes in TBST and TBS, blots were processed with Pierce ECL Plus reagent (ThermoFisher, Waltham, MA) and imaged using the Odyssey Fc imaging system (LI-COR Biosciences, Lincoln, NE). All blots are shown as single images with brightness and contrast adjusted to maximize visibility of the background and any non-specific bands.

### *In vitro* citrullination assays and anti-citrulline detection

Total protein (30 μg) from flies (larval or adult), bacteria, or a 1:1 mixture of each was aliquoted and diluted in extraction buffer to an equivalent final volume (up to 50 μL depending on the maximum SDS-PAGE well volume). PADs from bacteria were assayed as fusion proteins without cleavage of their N-terminal tags. Purified, recombinant human PAD2 was from Sigma-Aldrich (product #SAE0061). When assessing inhibition of PAD activity with Cl-amidine (CAS 1043444-18-3, Cayman Chemical, Ann Arbor, MI), the inhibitor (1 mM final, which is at least 50-fold over the IC50 for PADs) or ddH_2_O was added and all samples were incubated at 37 ºC for 30 minutes. To initiate enzymatic activity, CaCl_2_ (10 mM final) or ddH_2_O was added and samples were incubated at 37 ºC for 1 hour. After incubation, samples were quenched with EDTA (19 mM final). Samples were then mixed with Laemmli sample buffer and run on a 4–12% SDS-PAGE gel under reducing conditions. Proteins were transferred to PVDF membranes, which were then treated with an anti-Citrulline (Modified) Detection Kit (Millipore, Billerica, MA) reagent B (0.5% 2,3-butanedione monoxime, 0.25% antipyrine, and 0.5 M acetic acid) and Reagent A (0.025% FeCl_3_, 24.5% H_2_SO4, and 17% H_3_PO_4_) 1:1 mix inside of a Parafilm-sealed container and protected from light at 37 ºC for 3 hours. The membranes were rinsed with ddH_2_O and blocked in 5% milk/TBST buffer for 1 hour followed by a 2-hour incubation in 5% milk/TBST containing anti-citrulline (modified) human monoclonal antibody (1:1000 dilution) at room temperature with constant agitation. Membranes were subsequently incubated for 1 hour in 5% milk/TBST buffer containing a goat anti-human secondary antibody (1:2000 dilution) at room temperature with agitation. After washing with TBST and TBS, blots were processed and imaged as described above.

### Statistics

Behavioral studies were analyzed by one-way ANOVA with Tukey’s post hoc multiple comparison test. Bar graphs are expressed as the mean ± SEM; asterisks denote *p* ≤ 0.05. Survival data were compared by log-rank test using SigmaPlot 11.0 (Systat Software, San Jose, California).

## Results

### Development of transgenic PAD2 and PAD4 fly lines

**Fig 1:**
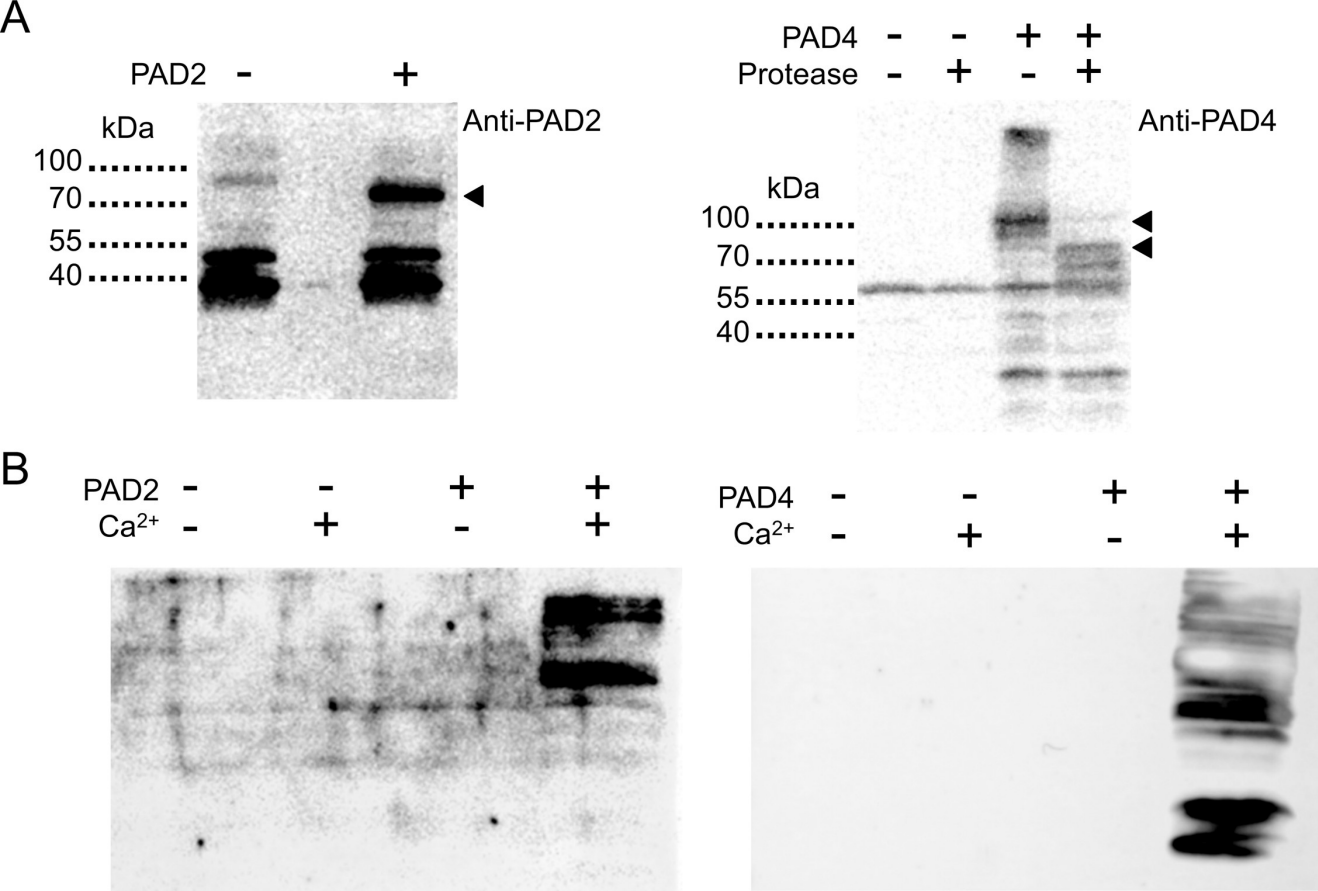
Human PAD2 and PAD4 expression in *E*. *coli* and *in vitro* citrullination activity. (A) Western blot for PAD2 (left) or PAD4 (right). *E*. *coli* were transformed with a PAD cDNA vector (+) or an empty vector control (-). Arrowheads point to bands specific to PAD-transformed *E*. *coli*. PAD4 was expressed as a GST-tagged fusion protein; the GST tag could be removed by addition of the appropriate protease. Non-specific labeling is also observed with both primary antibodies, based on the consistent bands seen in all lanes including the non-transformed controls. 10 μg of total protein was loaded per lane. (B) Citrullination activity of protein extracts from *E*. *coli* expressing PAD2 or PAD4, assessed in the presence (+) or absence (-) of 10 mM Ca^2+^. Citrullinated proteins were detected by Western blot.

To generate human PAD-expressing transgenic fly lines, plasmids harboring PAD2 or PAD4 cDNA were first validated by demonstrating recombinant expression in *E*. *coli* and *in vitro* PAD activity. Western blots for PAD2 or PAD4 from *E*. *coli* protein extracts showed specific bands not present in non-transformed bacteria (Fig 1A). These bands were located near the expected molecular weights for the PAD2 and PAD4 fusion proteins (78 and 101 kDa, respectively). Removal of the GST tag from PAD4 showed a band near that expected for the full-length protein (75 kDa) although smaller bands, possibly corresponding to truncation or degradation products, were also resolved (Fig 1A). Protein extracts showed citrullination activity only in the presence of supplemented Ca^2+^ (Fig 1B). The cDNA sequences of only the PAD ORFs were subsequently cloned into the pUAST vector to generate transgenic flies expressing PAD2 or PAD4 transgenes under the control of the GAL4/UAS system [43].

### PAD expression does not alter *Drosophila* lifespan or fecundity

**Fig 2:**
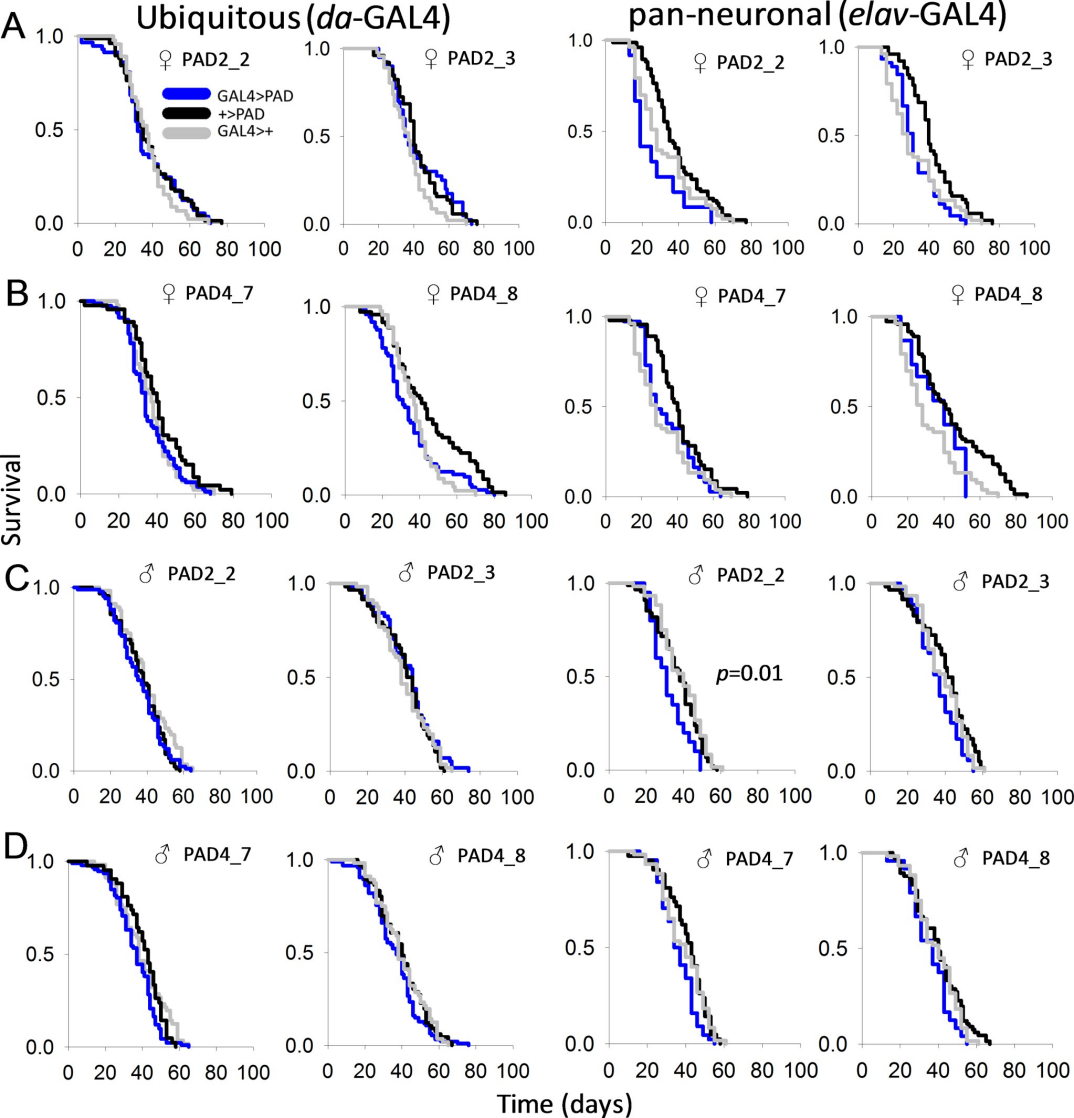
PAD overexpression does not alter lifespan in *D*. *melanogaster*. Ubiquitous (*da*-GAL4) or pan-neuronal (*elav*-GAL4) expression of (A) PAD2 or (B) PAD4 in female flies did not significantly affect lifespan. Similarly, overexpression of (C) PAD2 or (D) PAD4 did not consistently alter male survival. Control lines harbor only the GAL4 or the UAS-controlled PAD transgene. Two independent transgenic lines were assessed for each PAD (PAD2: PAD2_2 and PAD2_3; PAD4: PAD4_7 and PAD4_8). The log-rank *p* value is only shown when pairwise multiple comparisons reveal that survival curves differ between the experimental line and both controls.

We next determined if overexpression of PAD2 or PAD4 impacts fly lifespan. Ubiquitous expression of PAD2 or PAD4 using the *daughterless*-GAL4 (*da*-GAL4) driver line resulted in no significant differences in male or female survival compared to controls harboring only the PAD transgene or the GAL4 driver (Fig 2). Similar results were observed in two independent insertion lines for each PAD. PAD expression using the pan-neuronal *elav*-GAL4 driver, which induces broad expression in cells of the nervous system, also resulted in no consistent differences in survival (Fig 2).

**Fig 3:**
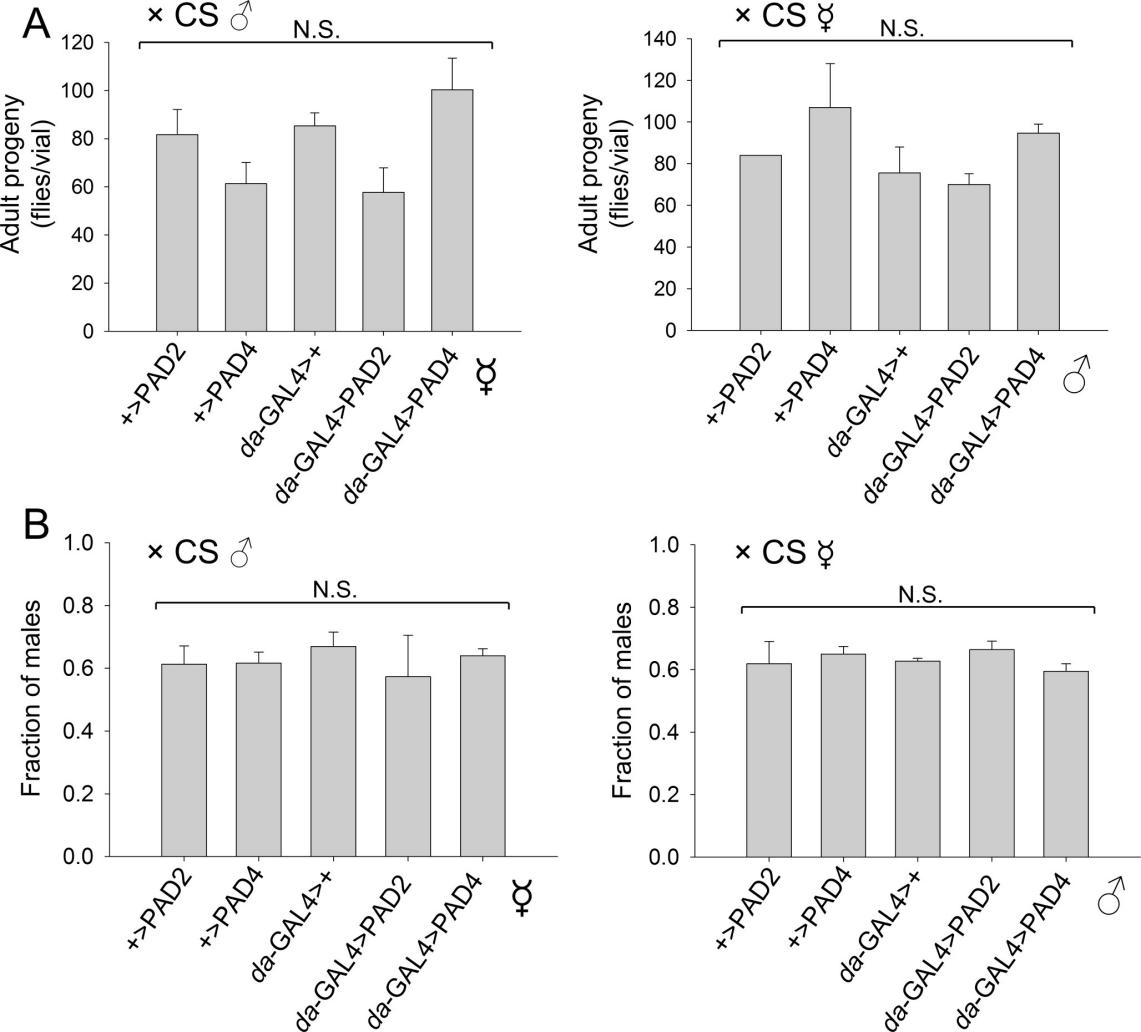
PAD overexpression does not alter fecundity. In reciprocal crosses of experimental flies with Canton-S (CS), fecundity was unaffected, as assessed by (A) the number of F1 progeny developed to adulthood and (B) the fraction of males in the F1 progeny.

To assess whether PAD expression affects fecundity, reciprocal crosses were established between PAD-expressing flies and Canton-S. There were no significant differences in the resulting number or sex ratio of progeny that reached adulthood from PAD-expressing and control parents (Fig 3). Hence, ubiquitous expression of PAD2 or PAD4 does not significantly affect reproductive performance.

### PAD expression does not affect sensitivity to acute heat stress

**Fig 4:**
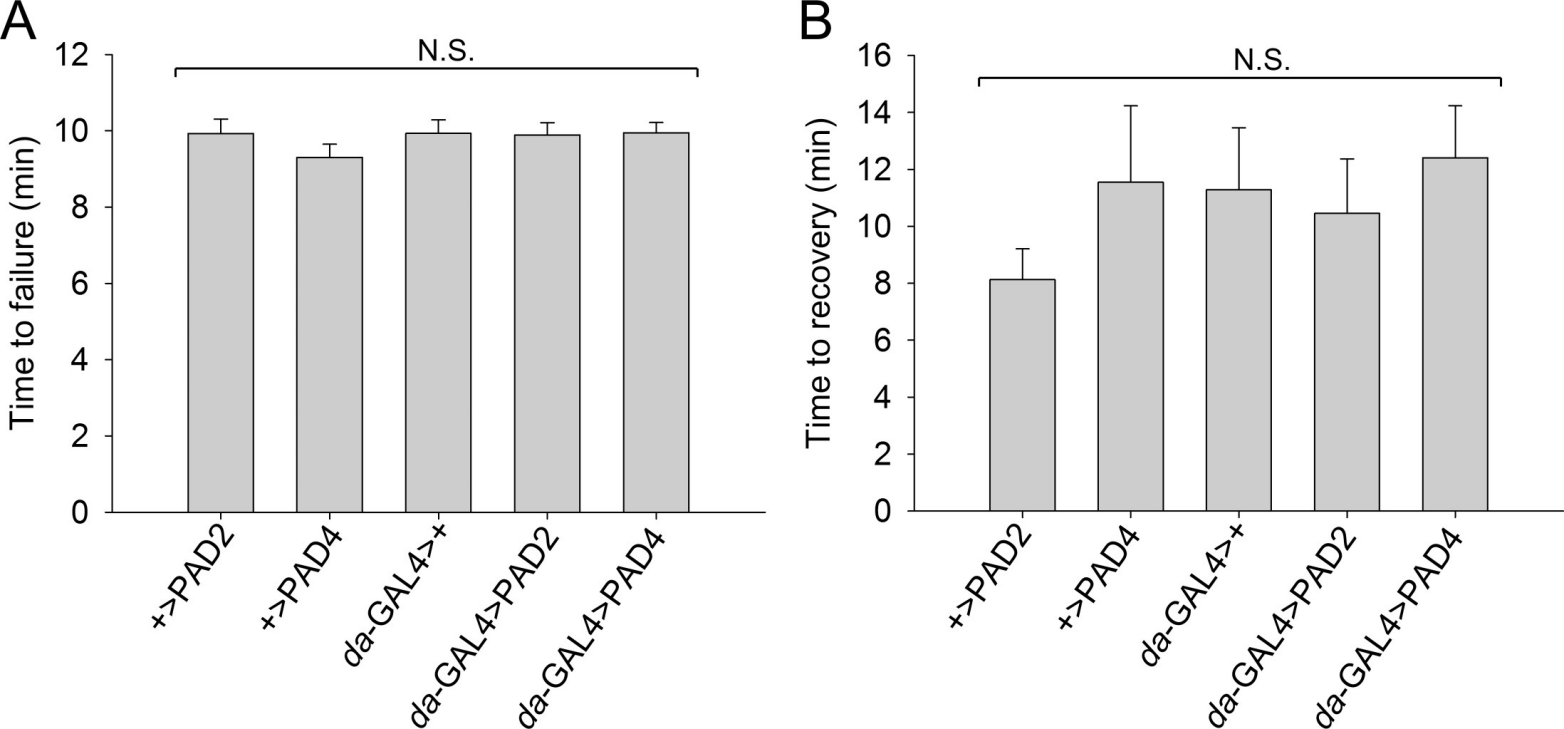
PAD overexpression does not affect the response to heat stress. (A) Average time to locomotor failure and (B) average time to recovery from acute hyperthermia was assessed in males (5–9 days old) individually housed in the *Drosophila* Activity Monitor.

Heat stress was previously shown to induce intracellular calcium signaling in flies [45]. Since PADs are activated by calcium [1], we speculated that PAD-expressing flies might be sensitive to thermal stress. Using locomotor failure as a readout, adult males ubiquitously expressing PAD2 or PAD4 were exposed to acute hyperthermia (40.5°C) and allowed to recover at room temperature. Regardless of genotype, over 90% of flies showed locomotor failure during the 12.5 minutes of heat treatment, and the time to locomotor failure did not differ between PAD-expressing files and their controls (Fig 4A). Similarly, recovery from heat, which was scored as the time required for restored locomotor function, was also not significantly different between PAD-expressing flies and their controls (Fig 4B).

### PAD expression and activity in transgenic flies

**Fig 5:**
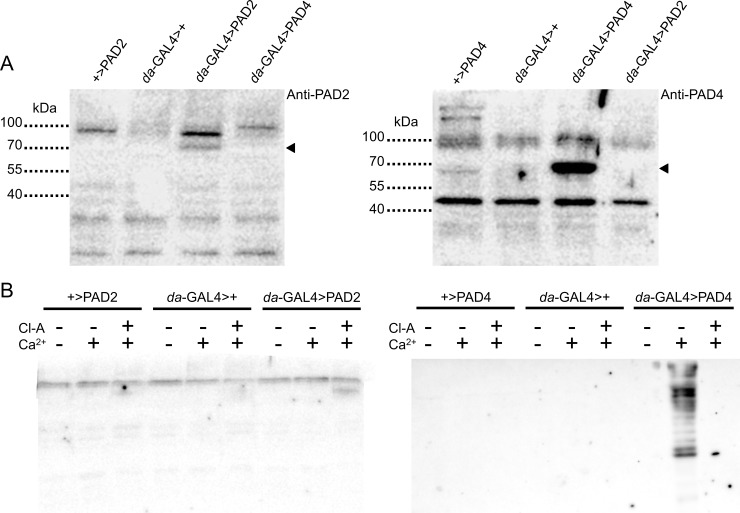
Human PAD2 and PAD4 expression and citrullination activity from *Drosophila* larvae. (A) Western blot for PAD2 (left) or PAD4 (right) using *Drosophila* larval protein extracts. Control lines harbor only the GAL4 or the UAS-controlled PAD transgene, while the experimental groups show PAD expression under the control of a ubiquitous GAL4 driver (*da*-GAL4). Arrowheads point to bands specific to PAD-expressing flies. Non-specific labeling is also observed with both primary antibodies, based on the consistent bands seen in all lanes including the negative controls. 30 μg of total protein was loaded per lane. (B) Detection of citrullinated proteins by Western blot of protein extracts from *Drosophila* larvae expressing PAD2 (left) or PAD4 (right). Cl-amidine (Cl-A, 1 mM) supplementation inhibits Ca^2+^-mediated PAD4 activity.

The lack of PAD-induced phenotypes in flies could be due to poor *in vivo* expression. To verify that transgenic lines driven by the ubiquitous promoter, *da*-GAL4, were expressing PADs, we assessed PAD expression by Western blotting of larval protein homogenates. Specific bands for PAD2 and PAD4 were clearly identified near the expected full-length molecular weights of 76 and 74 kDa, respectively (Fig 5A). We next probed for citrullinated proteins in larval homogenates. No citrullination was observed from control lines or from PAD-expressing flies without Ca^2+^ supplementation (Fig 5B). PAD4—but not PAD2—was active *in vitro* in the presence of Ca^2+^, and a potent PAD inhibitor, Cl-amidine [46], greatly reduced Ca^2+^-mediated PAD4 activity (Fig 5B).

### Human PAD2 expressed in *Drosophila* is inactive *in vitro*

**Fig 6:**
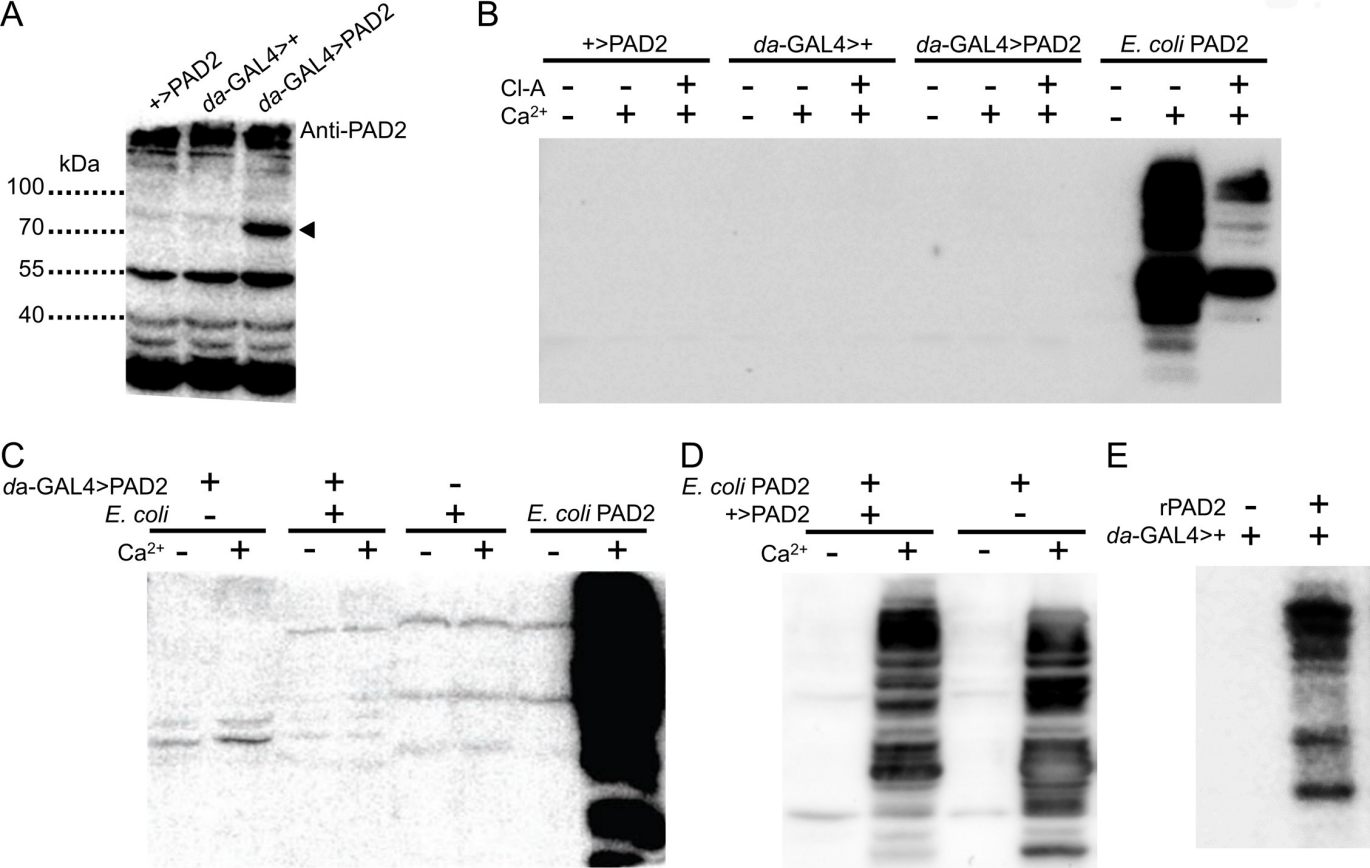
Human PAD2 expression and citrullination activity from *Drosophila* adults. (A) Western blot for PAD2 using fly protein extracts. Control lines harbor only the GAL4 or the UAS-controlled PAD transgene, while the experimental group shows PAD2 expression under the control of a ubiquitous GAL4 driver (*da*-GAL4). Arrowhead points to a band specific to PAD2-expressing flies. Non-specific labeling is also observed, based on the consistent bands seen in all lanes including the negative controls. 25 μg of total protein was loaded per lane. (B) Detection of citrullinated proteins by Western blot of protein extracts from *Drosophila* adults expressing PAD2. As a positive control, citrullination activity of a protein extract from PAD2-expressing *E*. *coli* is also shown. Cl-amidine (Cl-A, 1 mM) supplementation inhibits *E*. *coli*-expressed PAD2 activity. (C) Detection of citrullinated proteins by Western blot of protein extracts from flies ubiquitously expressing PAD2, supplemented with or without a protein extract from *E*. *coli*. As a positive control, citrullination activity of a protein extract from PAD2-expressing *E*. *coli* is also shown. (D) Detection of citrullinated proteins by Western blot of protein extracts from *E*. *coli* expressing PAD2 mixed with or without control fly homogenate (not expressing PAD2). (E) Detection of citrullinated proteins by Western blot of protein extracts from control flies (not expressing PAD2) with or without purified recombinant PAD2 (rPAD2).

To further investigate the potential enzymatic activity of fly-expressed PAD2, we examined protein expression in adult flies. PAD2 near the expected molecular weight (76 kDa) was found in protein extracts from *da*-GAL4>PAD2 flies (Fig 6A) but again, no enzymatic activity was detected *in vitro*, contrasting with the high citrullination activity (and Cl-amidine mediated inhibition) observed in extracts of PAD2-expressing *E*. *coli* (Fig 6B). To test whether flies might lack suitable PAD2 substrates, we applied fly-expressed PAD2 to a bacterial protein extract lacking PAD2. Fly and bacterial extracts were mixed 1:1 and supplemented with Ca^2+^ to induce citrullination. No enzymatic activity was observed in the mixture, or in bacterial or fly homogenates alone (Fig 6C). Consistent with our previous results, an extract of *E*. *coli* expressing PAD2 showed high Ca^2+^-induced citrullination.

We next tested the hypothesis that fly homogenates contain a specific inhibitor of PAD2 by mixing an extract from PAD2-expressing bacteria with fly homogenate lacking PAD2. Addition of Ca^2+^ to this mixture induces citrullination in a pattern that is different than bacterial homogenate alone (Fig 6D). Finally, we added commercially purified recombinant PAD2 to a fly homogenate lacking PAD2 and also observed citrullination of fly proteins (Fig 6E). These results are consistent with the idea that fly proteins can serve as PAD2 substrates and also suggest that the failure to observe PAD2 activity *in vivo* is not due to the presence of specific PAD2 inhibitors.

## Discussion

We have described the generation of human PAD2 and PAD4 overexpression models in *Drosophila* using the GAL4/UAS system for spatiotemporal control of gene expression [43]. Surprisingly, expression of PADs did not consistently alter lifespan or reproductive output. Although we verified PAD expression in *Drosophila* by Western blot, the lack of any organismal phenotypes is associated with an absence of citrullinated proteins from fly homogenates. Notably, we also find no citrullination in lysates of bacteria expressing PADs until Ca^2+^ is supplemented.

PAD activity, at least *in vitro*, requires supraphysiologic concentrations of Ca^2+^[47–49]. Consistent with this idea, the pathophysiology of several PAD-related diseases is linked to increased Ca^2+^ levels, loss of Ca^2+^ homeostasis, or mechanisms that enhance calcium sensitivity of the enzyme—thereby lowering Ca^2+^ requirements [31,50–52]. Thus, we hypothesized that fly-expressed PADs might exert activity *in vivo* if we could sufficiently mobilize Ca^2+^ stores. During normal physiological function at room temperature, cytosolic Ca^2+^ concentration in the *Drosophila* neuromuscular junction ranges from ~40 to 140 nM, and this range is elevated (~200 to 300 nM) during acute heat stress [45,53]. Although we did not observe any alteration in the response to acute hyperthermia in flies expressing PAD2 or PAD4, we note that the EC_50_ of Ca^2+^-mediated PAD activation is in the μM range [2,7,49] and we did not quantify whether the heat treatment successfully induced any citrullination *in vivo*.

Although we observed citrullination activity from fly-expressed PAD4 when supplemented with Ca^2+^ *in vitro*—and this activity was specifically inhibited by the PAD inhibitor, Cl-amidine—we were unable to detect activity from fly-expressed PAD2. We expressed the same PAD2 ORF in bacteria as a tagged fusion protein and the resulting protein was shown to be active. Hence, we tested two hypotheses to potentially explain the lack of citrullination activity from fly-expressed PAD2: 1) flies may not have suitable protein substrates for PAD2 and 2) flies may harbor PAD2-specific inhibitors. By performing experiments mixing fly- and bacteria-derived PAD2, we ruled out both hypotheses and conclude that fly-expressed PAD2 is inactive, even though the expressed protein appears to be full length and DNA sequencing reveals an intact PAD2 ORF. Altered protein folding, cellular localization, or post-translational modifications such as proteolytic processing might explain the differences in Ca^2+^-mediated catalytic activity between fly- and *E*. *coli*-expressed PAD2. Interestingly, a previous study examining PAD1-3 from chicken also observed Ca^2+^-dependent catalytic activity from PAD1 and PAD3 expressed in insect cells, but not from PAD2—even after a conserved active site from mammalian PAD2 was restored [6]. Additionally, *Padi2* knockout mice that lack PAD2 protein do not display an apparent phenotype under normal physiological conditions [54].

Much is still unknown about the regulation of these enzymes and it has been suggested that co-factors other than Ca^2+^ may enable PADs to function at physiological Ca^2+^ concentrations [55,56]. Future studies might use the *Drosophila* ectopic expression system for human PADs to examine mechanisms of enzyme activation and to investigate the involvement of PADs in molecular pathways important in both normal cellular processes and in disease.

## Supporting information

S1 Raw Images (PDF)
