# Supplementary material for: In vivo expression of peptidylarginine deiminase in Drosophila melanogaster
Source: PLoS One. 2020 Jan 15;15(1):e0227822. doi: 10.1371/journal.pone.0227822 (PMC6961906; doi:10.1371/journal.pone.0227822)
Supplement: S1 Raw Images — (PDF) [file pone.0227822.s001.pdf]

Figure 1A - Imaged Western blots

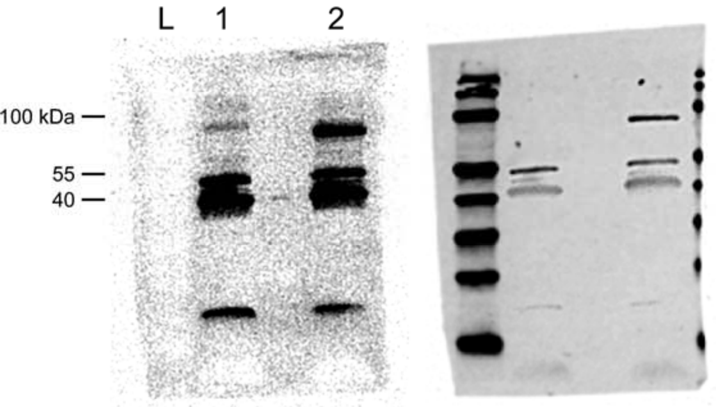


Anti-PAD2 blot
L = ThermoFisher PageRuler Prestained Protein Ladder
1 = *E. coli* negative control
2 = *E. coli* expressing PAD2
No sample was loaded in the lane between 1 and 2

On the right, the same blot at lower exposure is superimposed with the channel visualizing the ladder.

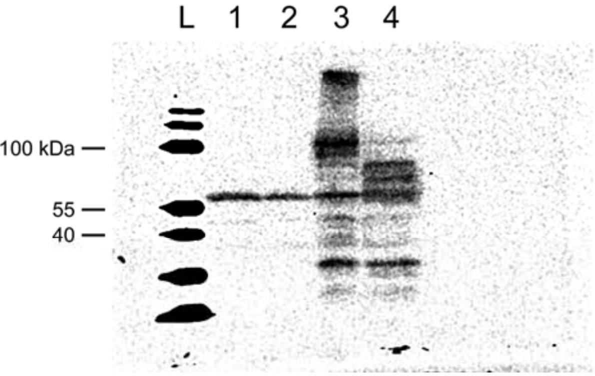


Anti-PAD4 blot
L = ThermoFisher PageRuler Prestained Protein Ladder
1 = *E. coli* negative control
2 = *E. coli* negative control treated with protease
3 = *E. coli* expressing PAD4
4 = *E. coli* expressing PAD4 treated with protease

Figure 1B - Imaged anti-citrulline blots

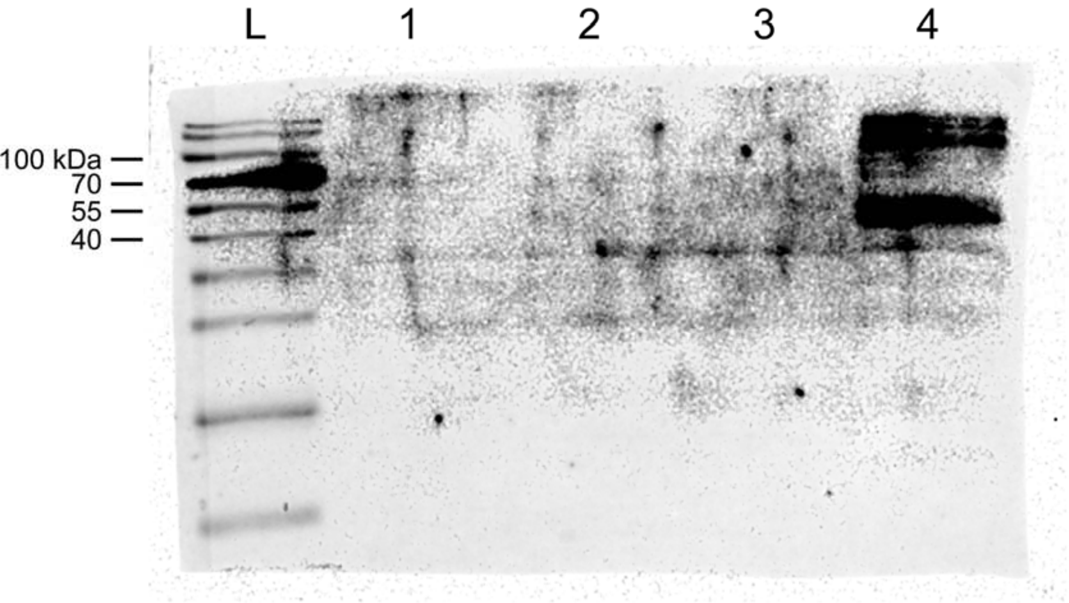


L = ThermoFisher PageRuler Prestained Protein Ladder
1 = *E. coli* negative control
2 = *E. coli* negative control supplemented with $Ca^{2+}$
3 = *E. coli* expressing PAD2
4 = *E. coli* expressing PAD2 supplemented with $Ca^{2+}$

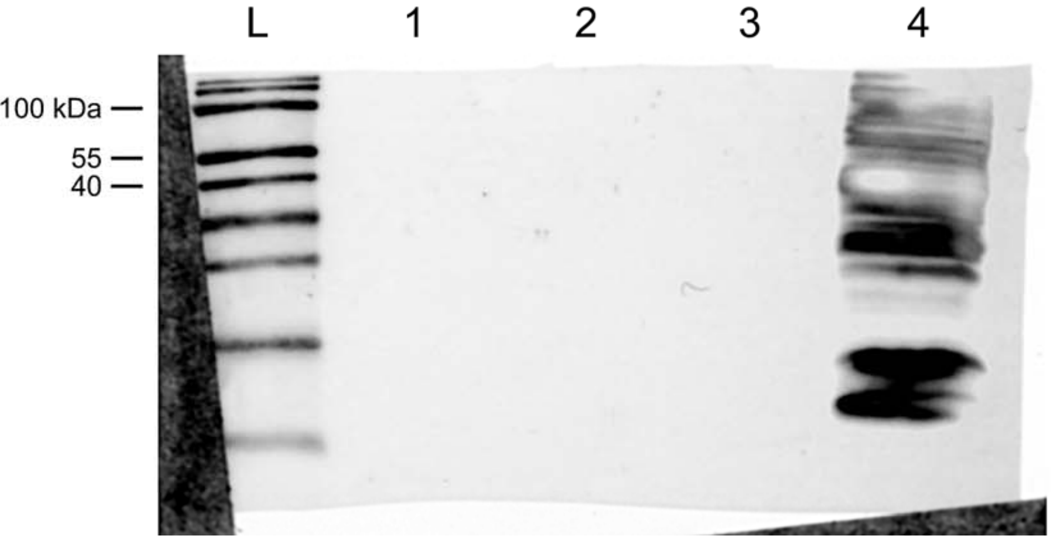


L = ThermoFisher PageRuler Prestained Protein Ladder
1 = *E. coli* negative control
2 = *E. coli* negative control supplemented with $Ca^{2+}$
3 = *E. coli* expressing PAD4
4 = *E. coli* expressing PAD4 supplemented with $Ca^{2+}$

Figure 5A - Imaged Western blots

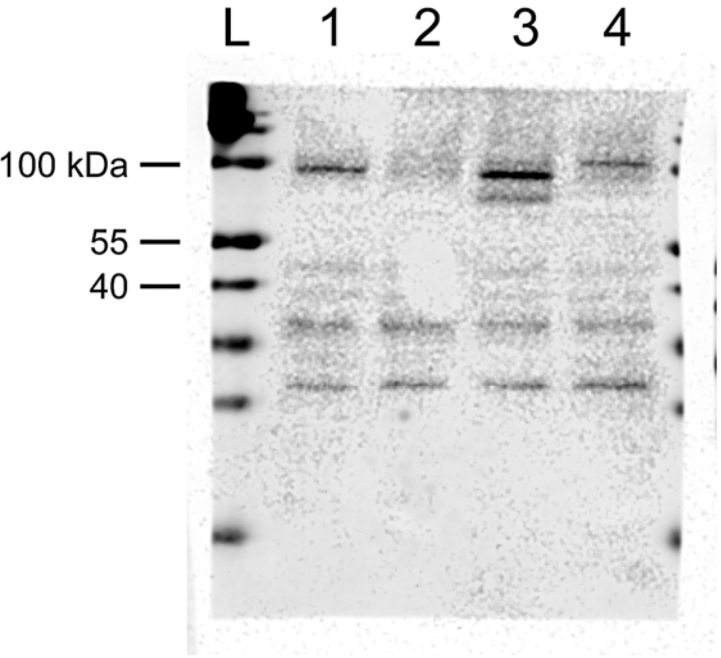


Anti-PAD2 blot
L = ThermoFisher PageRuler Prestained Protein Ladder
1 = *Drosophila* negative control (+>PAD2)
2 = *Drosophila* negative control (*da*-GAL4>+)
3 = *Drosophila* expressing PAD2 (*da*-GAL4>PAD2)
4 = *Drosophila* expressing PAD4 (*da*-GAL4>PAD4)

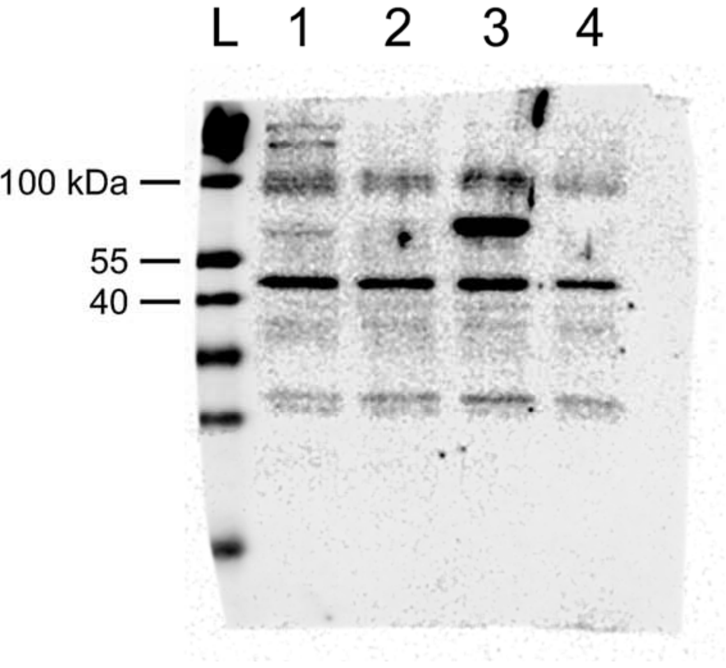


Anti-PAD4 blot
L = ThermoFisher PageRuler Prestained Protein Ladder
1 = *Drosophila* negative control (+>PAD4)
2 = *Drosophila* negative control (*da*-GAL4>+)
3 = *Drosophila* expressing PAD2 (*da*-GAL4>PAD4)
4 = *Drosophila* expressing PAD4 (*da*-GAL4>PAD2)

Figure 5B - Imaged anti-citrulline blots

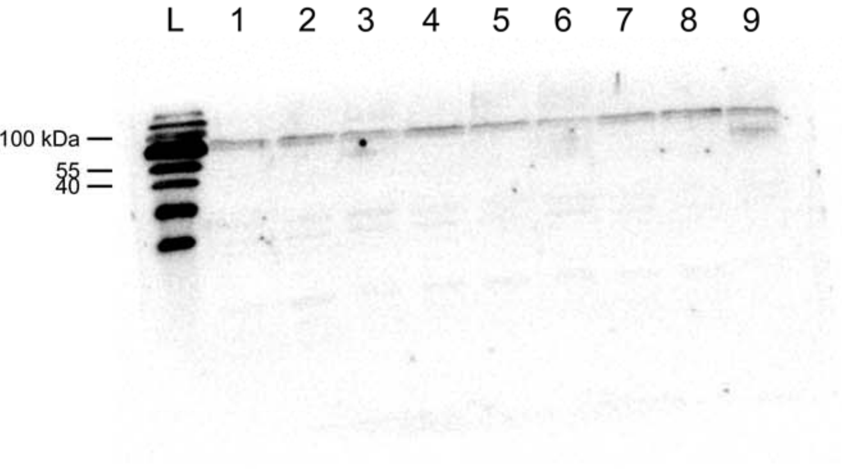


L = ThermoFisher PageRuler Prestained Protein Ladder
1 = *Drosophila* negative control (+>PAD2)
2 = *Drosophila* negative control (+>PAD2) supplemented with $Ca^{2+}$
3 = *Drosophila* negative control (+>PAD2) supplemented with $Ca^{2+}$ and Cl-A
4 = *Drosophila* negative control (*da*-GAL4>+)
5 = *Drosophila* negative control (*da*-GAL4>+) supplemented with $Ca^{2+}$
6 = *Drosophila* negative control (*da*-GAL4>+) supplemented with $Ca^{2+}$ and Cl-A
7 = *Drosophila* expressing PAD2 (da-GAL4>PAD2)
8 = *Drosophila* expressing PAD2 (da-GAL4>PAD2) supplemented with $Ca^{2+}$
9 = *Drosophila* expressing PAD2 (da-GAL4>PAD2) supplemented with $Ca^{2+}$ and Cl-A

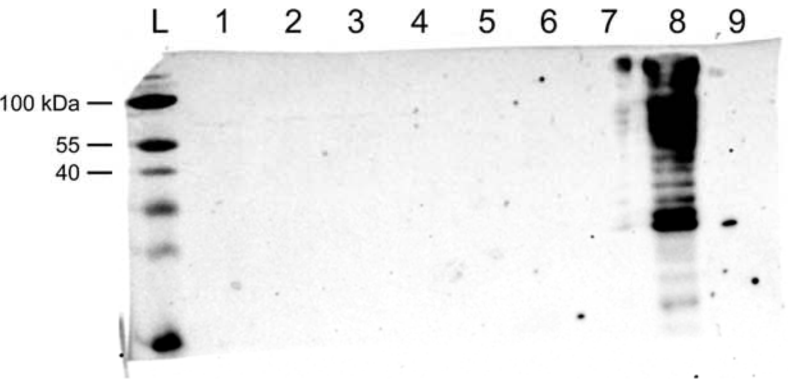


L = ThermoFisher PageRuler Prestained Protein Ladder
1 = *Drosophila* negative control (+>PAD4)
2 = *Drosophila* negative control (+>PAD4) supplemented with $Ca^{2+}$
3 = *Drosophila* negative control (+>PAD4) supplemented with $Ca^{2+}$ and Cl-A
4 = *Drosophila* negative control (*da*-GAL4>+)
5 = *Drosophila* negative control (*da*-GAL4>+) supplemented with $Ca^{2+}$
6 = *Drosophila* negative control (*da*-GAL4>+) supplemented with $Ca^{2+}$ and Cl-A
7 = *Drosophila* expressing PAD2 (da-GAL4>PAD4)
8 = *Drosophila* expressing PAD2 (da-GAL4>PAD4) supplemented with $Ca^{2+}$
9 = *Drosophila* expressing PAD2 (da-GAL4>PAD4) supplemented with $Ca^{2+}$ and Cl-A

Figure 6A - Imaged Western blot

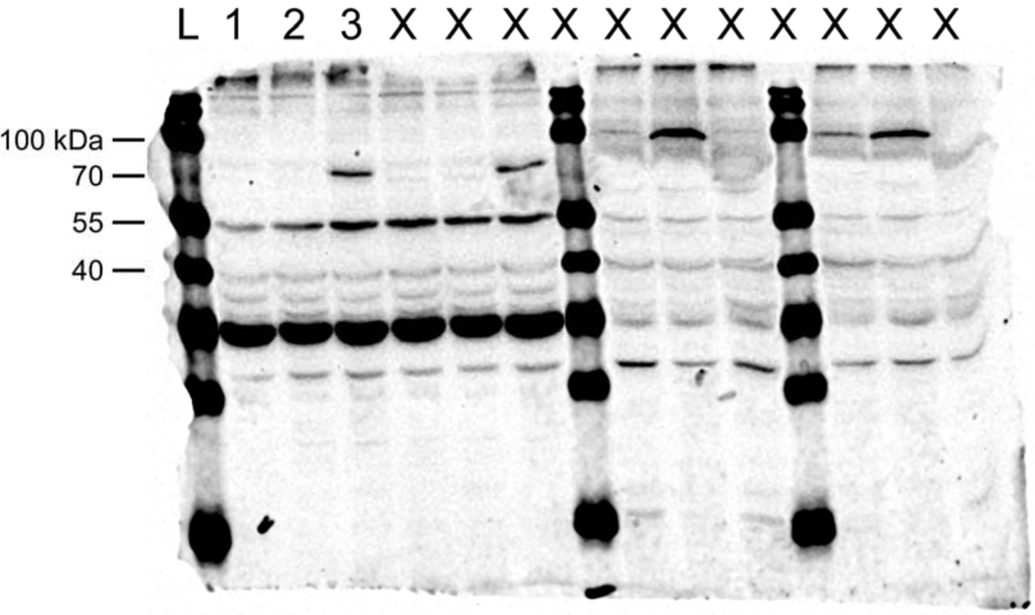


Anti-PAD2 blot
L = ThermoFisher PageRuler Prestained Protein Ladder
1 = *Drosophila* negative control (+>PAD2)
2 = *Drosophila* negative control (*da*-GAL4>+)
3 = *Drosophila* expressing PAD2 (*da*-GAL4>PAD2)

Figure 6B - Imaged anti-citrulline blot

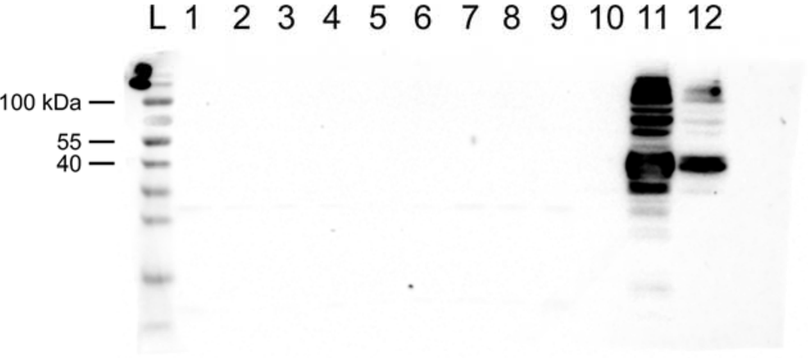


L = ThermoFisher PageRuler Prestained Protein Ladder
1 = *Drosophila* negative control (+>PAD2)
2 = *Drosophila* negative control (+>PAD2) supplemented with $Ca^{2+}$
3 = *Drosophila* negative control (+>PAD2) supplemented with $Ca^{2+}$ and Cl-A
4 = *Drosophila* negative control (*da*-GAL4>+)
5 = *Drosophila* negative control (*da*-GAL4>+) supplemented with $Ca^{2+}$
6 = *Drosophila* negative control (*da*-GAL4>+) supplemented with $Ca^{2+}$ and Cl-A
7 = *Drosophila* expressing PAD2 (da-GAL4>PAD2)
8 = *Drosophila* expressing PAD2 (da-GAL4>PAD2) supplemented with $Ca^{2+}$
9 = *Drosophila* expressing PAD2 (da-GAL4>PAD2) supplemented with $Ca^{2+}$ and Cl-A
10 = *E. coli* expressing PAD2
11 = *E. coli* expressing PAD2 supplemented with $Ca^{2+}$
12 = *E. coli* expressing PAD2 supplemented with $Ca^{2+}$ and Cl-A

Figure 6C - Imaged anti-citrulline blot

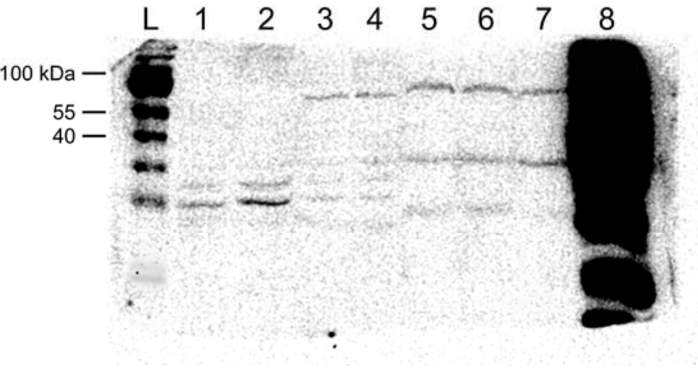


L = ThermoFisher PageRuler Prestained Protein Ladder
1 = *Drosophila* expressing PAD2 (da-GAL4>PAD2)
2 = *Drosophila* expressing PAD2 (da-GAL4>PAD2) supplemented with $Ca^{2+}$
3 = *Drosophila* expressing PAD2 (da-GAL4>PAD2) supplemented with *E. coli* protein extract
4 = *Drosophila* expressing PAD2 (da-GAL4>PAD2) supplemented with *E. coli* protein extract and $Ca^{2+}$
5 = *E. coli* protein extract
6 = *E. coli* protein extract supplemented with $Ca^{2+}$
7 = *E. coli* expressing PAD2
8 = *E. coli* expressing PAD2 supplemented with $Ca^{2+}$

Figure 6D - Imaged anti-citrulline blot

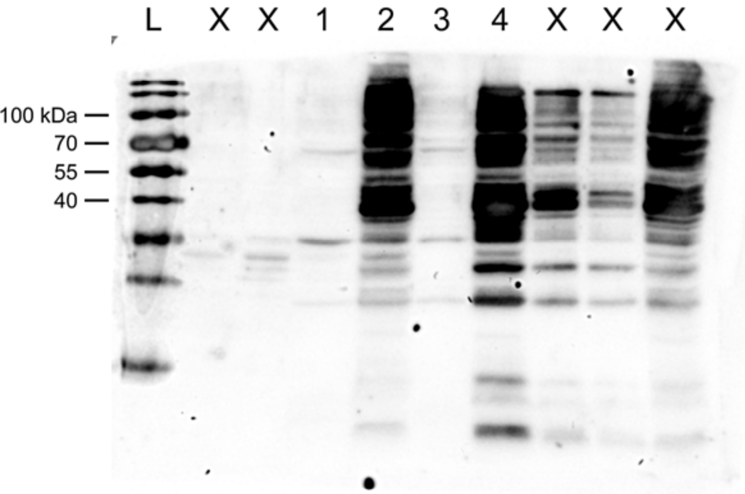


L = ThermoFisher PageRuler Prestained Protein Ladder

1 = *E. coli* expressing PAD2 supplemented with *Drosophila* protein extract (+>PAD2)

2 = *E. coli* expressing PAD2 supplemented with *Drosophila* protein extract (+>PAD2) and $Ca^{2+}$

3 = *E. coli* expressing PAD2

4 = *E. coli* expressing PAD2 supplemented with $Ca^{2+}$

Figure 6E - Imaged anti-citrulline blot

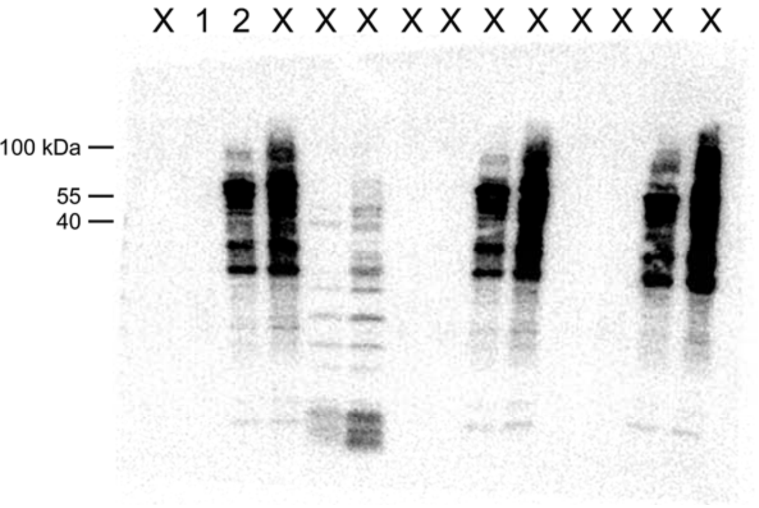


1 = *Drosophila* protein extract (*da*-GAL4>+)
2 = *Drosophila* protein extract (*da*-GAL4>+) supplemented with purified, recombinant PAD2
